# Glucocorticoids alleviate particulate matter-induced COX-2 expression and mitochondrial dysfunction through the Bcl-2/GR complex in A549 cells

**DOI:** 10.1038/s41598-023-46257-y

**Published:** 2023-11-02

**Authors:** Yeon-Ji Park, June Heo, Yonghyeon Kim, Hyeseong Cho, Myeongkuk Shim, Kyunghyun Im, Wonchung Lim

**Affiliations:** 1 Department of Biochemistry and Molecular Biology, Ajou University School of Medicine, Suwon, Republic of Korea; 2 Department of Biomedical Sciences, Graduate School of Ajou University, Suwon, Republic of Korea; 3 BL Healthcare, Yongin-si, Gyeonggi-do 16827 South Korea; 4 Department of Sports Medicine, College of Health Science, Cheongju University, Cheongju, 28503 South Korea

**Keywords:** Environmental sciences, Endocrinology

## Abstract

Exposure to particulate matter (PM) causes mitochondrial dysfunction and lung inflammation. The cyclooxygenase-2 (COX-2) pathway is important for inflammation and mitochondrial function. However, the mechanisms by which glucocorticoid receptors (GRs) suppress COX-2 expression during PM exposure have not been elucidated yet. Hence, we examined the mechanisms underlying the dexamethasone-mediated suppression of the PM-induced COX-2/prostaglandin E2 (PGE2) pathway in A549 cells. The PM-induced increase in COX-2 protein, mRNA, and promoter activity was suppressed by glucocorticoids; this effect of glucocorticoids was antagonized by the GR antagonist RU486. COX-2 induction was correlated with the ability of PM to increase reactive oxygen species (ROS) levels. Consistent with this, antioxidant treatment significantly abolished COX-2 induction, suggesting that ROS is involved in PM-mediated COX-2 induction. We also observed a low mitochondrial membrane potential in PM-treated A549 cells, which was reversed by dexamethasone. Moreover, glucocorticoids significantly enhanced Bcl-2/GR complex formation in PM-treated A549 cells. Glucocorticoids regulate the PM-exposed induction of COX-2 expression and mitochondrial dysfunction and increase the interaction between GR and Bcl-2. These findings suggest that the COX-2/PGE2 pathway and the interaction between GR and Bcl-2 are potential key therapeutic targets for the suppression of inflammation under PM exposure.

## Introduction

The World Health Organization (WHO) has reported that particulate matter (PM) is a common indicator of air pollution, and PM_2.5_ can penetrate the lung barrier and enter the blood system (WHO; https://www.who.int). Several studies have reported that PM_2.5_ exposure can increase mortality from respiratory disease and lung cancer^1,2^. Burnett et al. showed that the relationships between PM concentration and chronic obstructive pulmonary disease mortality are non-linear, with mortality risks increasing rapidly at low PM_2.5_ levels and reaching a plateau at high levels^3^. Data from in vivo and in vitro experiments have shown that PM_2.5_ can cause mitochondrial dysfunction of lung cells and even induce acute and chronic lung inflammation^4–8^. PM-induced apoptosis and inflammation are mediated by cyclooxygenase-2 (COX-2)^9^.

Mitochondria play a vital role in energy metabolism in the cell. PM can induce mitochondrial imbalances by increasing mitochondrial permeability transition pore opening, decreasing respiration capacity^10^, and reducing the mitochondrial membrane potential (MMP)^11^. It has been proved that PM can cause airway inflammation and lung diseases by mitochondrial dysfunction^12,13^. Inflammation is triggered by dysfunctional mitochondria, represented by the induction of COX-2^14^. The selective COX-2 inhibitor parecoxib improves mitochondrial function by reducing prostaglandin E2 (PGE2)^15^. Mitochondrial-located COX-2 protects tumor cells via impairing mitochondrial damage and apoptosis^16^. Thus, mitochondria could be an important target for lung diseases development and mechanisms.

Functional glucocorticoid receptors (GRs) play an important role in normal lung development, and glucocorticoids suppress acute lung injury via GR dimerization^17,18^. Mitochondrial GR has some effects on mitochondrial inflammation^19^. Jia et al. reported that PM exposure inhibited GR activation and induced cytokine activation in mice^20^. The N-terminal transactivation domain of the GR is critical for apoptosis in small-cell lung cancer (SCLC)^21^. GR-induced apoptosis in SCLC requires protein interaction with GRs and Bcl-2^22^. PM aggravates mitochondrial damage via downregulation of Bcl-2^23^. GR forms a complex with Bcl-2 and translocates Bcl-2 to the mitochondria^24^. COX-2’s antiapoptotic effect is related to Bcl-2^25^. Mitochondrial COX-2 might be involved in regulating Bcl-2 to maintain mitochondrial integrity^26^. GR modulates air pollution-induced lung injury and inflammation^27^. Thus, PM-mediated cellular events may be associated with GR signaling. PM-induced mitochondrial dysfunction and GR signaling via the mitochondria may converge to regulate the lung inflammatory response. Dexamethasone inhibited COX-2 induction in a GR-dependent manner^28,29^.

A549 cells are widely used as models of pulmonary epithelial cells. However, the mechanism by which GR suppresses PM-induced COX-2 expression in A549 cells has not been examined. This study investigated how GR suppresses PM-induced COX-2 expression via the mitochondrial pathway in A549 cells.

## Material and methods

### Cell culture and treatment

Human pulmonary epithelial A549 (ATCC, CCL-185) cells were maintained in DMEM containing 10% FBS and penicillin/streptomycin. Cells were grown at 37 ℃ in a humidified atmosphere of 95% air/5% CO2 and fed every 2–3 days. Before treatment, the cells were washed with phosphate-buffered saline and cultured in DMEM/5% charcoal–dextran stripped FBS (CD-FBS) for 2 days. All treatments were done with DMEM/5% CD-FBS. The PM is Standard Reference Material 1648a (National Institute of Standards and Technology, United States). SRM 1648a has 25 metals, 21 polycyclic aromatic hydrocarbons, and 7 polychlorinated biphenyls. Cells were treated for 24 h with PM (300 µg/ml) in the absence or presence of dexamethasone (0.1 µM) and/or RU486 (1 µM).

### Plasmids

COX-2-Luc, a firefly luciferase reporter construct containing the human COX-2 gene promoter fragment − 327/+ 59, was kindly provided by Dr. Hiroyasu Inoue (Nara Women’s University, Nara, Japan).

### Transfection and luciferase assays

A549 cells were transiently transfected with plasmids by using the polyethylenimine (PEI; Polysciences, Warrington, PA, USA). Luciferase activity was determined 24 or 48 h after treatment with an AutoLumat LB9507 luminometer (EG & G Berthold, Bad Widbad, Germany) using the luciferase assay system (Promega Corp., Madison, WI, USA) and expressed as relative light units.

### Reverse transcription (RT)-PCR

Total RNA was extracted using Trizol Reagent according to the manufacturer's instruction. RNA pellets were dissolved in diethylpyrocarbonate-treated water. The yield of RNA was quantified by spectroscopy at 260 nm. Samples were aliquoted and stored at − 80 °C until further processing. To synthesize first strand cDNA, 3 μg total RNA was incubated at 70 °C for 5 min with 0.5 μg of random hexamer and deionized water (up to 11 μl). The RT reaction was performed using 40 U of M-MLV reverse transcriptase (Promega) in 5 × reaction buffer (250 mmol l^−1^ Tris–HCl; pH 8.3, 375 mM KCl, 15 mM MgCl_2_, 50 mM DTT), RNase inhibitor at 1 U μl^−1^, and 1 mM dNTP mixtures at 37 °C for 60 min. Quantitative real-time PCR (qPCR) was performed using iQ™ SYBR Green Supermix (Bio-Rad, Hercules, CA, USA). The primers used were: β-actin sense primer, 5′-CAAATGCTTCTAGGCGGACTATG-3′; β-actin anti-sense primer, 5′-TGCGCAAGTTAGGTTTTGTCA-3′; COX-2 sense primer, 5′-TGAAGAACTTACAGGAGAAAA-3′; COX-2 anti-sense primer, 5′-TACCAGAAGGGCAGGATACA-3′. A final volume was 25 μl, and an iCycler iQ Real-time PCR Detection System (Bio-Rad) was used for qPCR. The amplification data were analysed by iQ™5 optical system software version 2.1 and calculated using the ΔΔCT method. The ΔΔCT method was used to calculate relative mRNA expression.

### Western blot analysis

Protein was isolated in lysis buffer (150 mM NaCl, 50 mM Tris–HCl, 5 mM EDTA, 1% Nonidet P-40, 0.5% deoxycholate, 1% SDS) with protease inhibitor cocktail (Sigma, St. Louis, MO, USA) on ice for 1 h and then centrifuged for 20 min at 13,000×*g*. Supernatant was collected and protein concentrations were measured using the Bradford method (Bio-Rad, Hercules, CA, USA). Proteins were dissolved in sample buffer and boiled for 5 min prior to loading onto an acrylamide gel. After SDS-PAGE, proteins were transferred to a polyvinylidene difluoride membrane, blocked with 5% nonfat dry milk in Tris-buffered saline containing 0.1% Tween-20 (TBST) for 60 min at room temperature. The membranes were incubated for 2 h at room temperature with antibody. Equal lane loading was assessed using actin monoclonal antibody (Sigma, St. Louis, MO, USA). After washing with TBST, blots were incubated with 1:5000 dilution of the horseradish peroxidase conjugated-secondary antibody (Invitrogen, Grand Island, NY, USA), and washed again three times with TBST. The transferred proteins were visualized with an enhanced chemiluminescence detection kit (Amersham Pharmacia Biotech, Buckinghamshire, UK).

### Immunoprecipitation

Five hundred micrograms of the cell lysates were mixed with 1 μg of antibody and incubated overnight at 4 °C with constant rotation. To recover immunoprecipitated complexes, 50 μl of protein A-sepharose, diluted 1:1 in PBS, were then added to the samples and incubated on ice. The beads were pelleted by centrifugation and the bound proteins were eluted by incubation in 2 × SDS loading buffer by boiling. The eluted proteins were analysed by immunoblot analysis.

### Analysis of intracellular ROS levels by flow cytometry

Intracellular ROS levels were determined by staining cells with 10 mM of dichlorofluorescin diacetate (DCF-DA) fluorescence dye (Molecular Probes) for 20 min. Cells were collected by trypsinization, and the fluorescent intensities were quantified by flow cytometry (FACS Vantage, Becton Dickinson).

### Quality and membrane integrity of isolated mitochondria

Quality of isolated mitochondria and the integrity of membrane were assessed using the JC-1 test by Mitochondria Staining Kit (CS0390, Sigma, USA).

### Microscopy analysis

For visualization of mitochondria, cells were stained for 30 min with 125 nM MitoTracker Red™. For immunofluorescence staining, cells were fixed with methanol/acetone (1:1) solution, pre-incubated in blocking solution, followed by incubation with primary antibodies at 4 °C, and probed with fluorescence-conjugated antibody. Images were captured under confocal microscopy (Nikon A1R).

### Determination of PGE2

PGE2 production in PM-treated A549 cells was measured using an ELISA kit according to the manufacturer’s instructions (514010, Cayman, USA).

### Statistical analysis

Data were expressed as means ± SE, and group comparisons were made by one-way ANOVA. The Tukey–Kramer method was used as a post hoc test when ANOVA reached significance (*P* < 0.05).

## Results

### PM induces COX-2 expression, and GR suppresses PM-induced COX-2 expression in A549 cells

**Figure 1:**
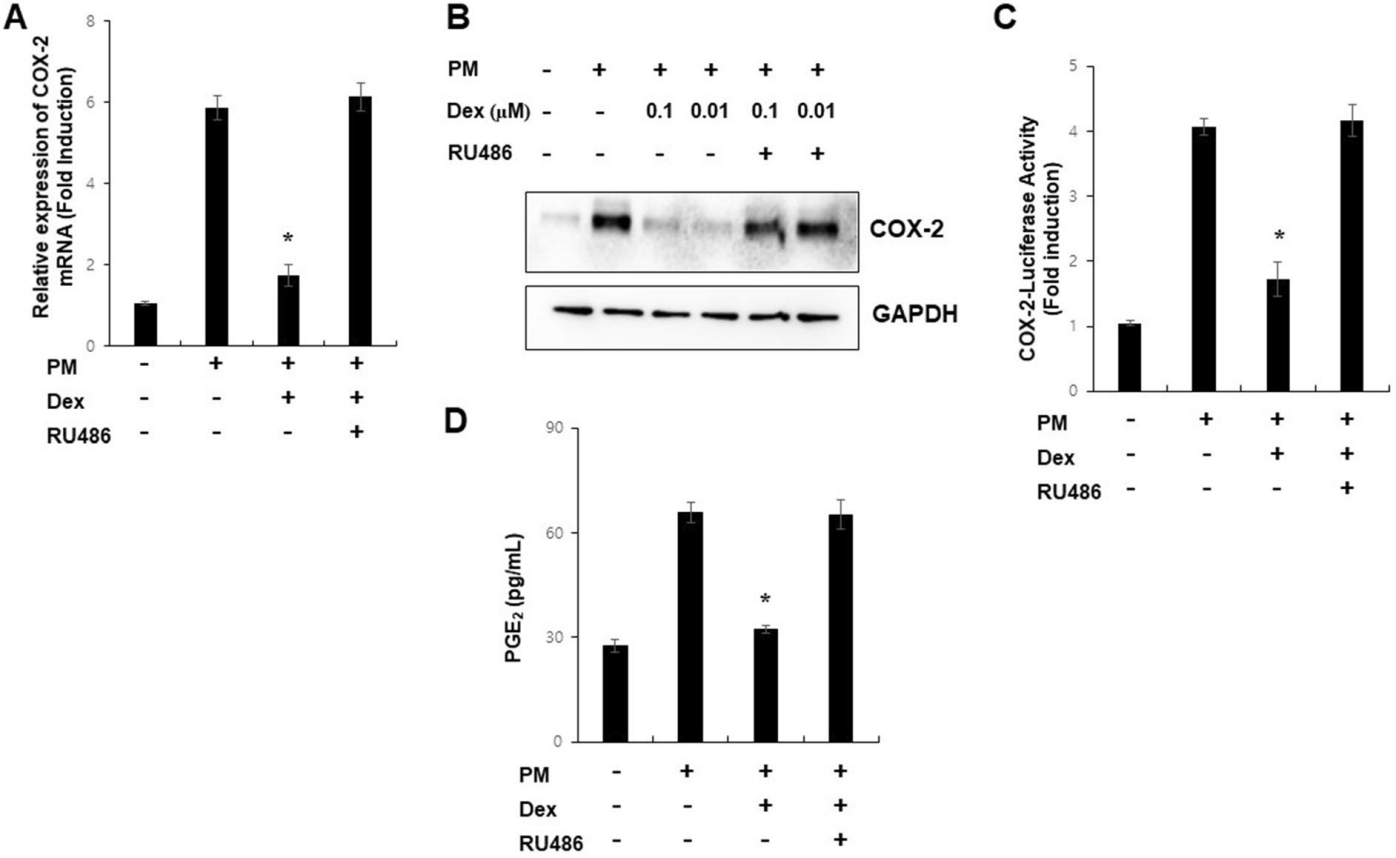
PM induces COX-2 expression, and GR suppresses PM-induced COX-2 expression in A549 cells. (**A**) A549 cells were treated with PM (300 µg/ml) as indicated for 24 h and analyzed by qPCR. (**B**) A549 cells were pretreated with dexamethasone (0.1 µM) and/or RU486 (1 µM) for 1 h before treatment with PM for 24 h and analyzed by Western blot. (**C**) A549 cells were transfected with COX-2-Luc and treated as indicated. After treatment, luciferase expression was determined. (**D**) The production of PGE2 was measured. Values represent the mean ± SEM (N = 3). *P < 0.05. All experiments were repeated three times. The images of the original blots are available in Supplementary Fig. 1.

COX-2 expression is induced by PM and has been implicated in lung inflammation^30^ and metastasis^31^. However, there are no reports on GR-mediated suppression of PM-induced COX-2 expression in lung alveolar epithelial cells. As an experimental control for GR, we performed an GRE-dependent reporter gene assay in our system (Supplementary Fig. 4). To examine the role of GR in PM-mediated induction of COX-2 expression by PM in A549 cells, we pre-incubated cells with glucocorticoids and/or RU486 for 1 h and co-treated them with PM. Dexamethasone blocked PM-induced COX-2 mRNA and protein expression, COX-2 promoter activity, and prostaglandin E2 production (Fig. 1). Hypertonic sodium chloride induction of COX-2 is used for positive control (Supplementary Fig. 5). This suppressive effect of glucocorticoids was antagonized by RU486 or GR knock-down (Supplementary Fig. 3), suggesting that GR mediates this regulation. These results indicated that glucocorticoids inhibited PM-induced COX-2 expression via GRs in A549 cells.

### GR suppresses PM-mediated induction of COX-2 by influencing mitochondrial ROS production

**Figure 2:**
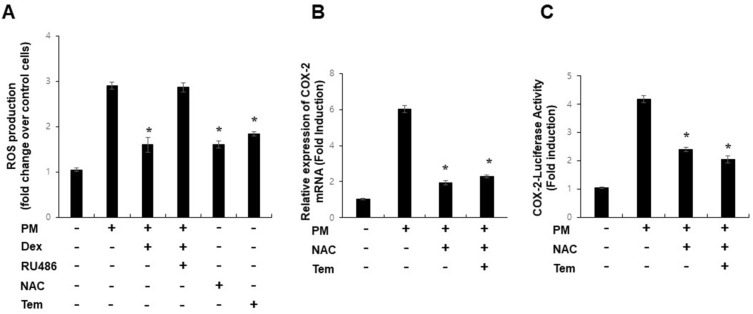
GR suppresses PM-mediated induction of COX-2 by influencing mitochondrial ROS production. (**A**) Intracellular ROS levels were determined by incubation of cells with 10 mM DCF-DA for 20 min followed by flow cytometry. (**B**) A549 cells were treated with PM (300 µg/ml) as indicated for 24 h and analyzed by qPCR. (**C**) A549 cells were transfected with COX-2-Luc and treated as indicated. After treatment, luciferase expression was determined. Values represent the mean ± SEM (N = 3). *P < 0.05. All experiments were repeated three times.

PM-induced lung inflammation is associated with oxidative stress^13,31^. GR regulates inflammatory signaling by suppressing ROS^32^. Therefore, we investigated whether GR affected PM-mediated mitochondrial ROS production. ROS levels were increased by PM treatment, and dexamethasone blocked PM-mediated mitochondrial ROS production (Fig. 2A), indicating a close correlation between COX-2 suppression and ability of GR to inhibit ROS. When PM-treated cells were incubated with antioxidants, N-acetyl cysteine, or tempol, the induction of COX-2 mRNA expression and COX-2 promoter activity by PM was significantly blocked (Fig. 2B,C). Thus, these results suggest that PM-induced COX-2 expression is mediated by mitochondrial ROS production and that GR can inhibit PM induction via ROS.

### Glucocorticoids alleviate PM-induced mitochondrial dysfunction in A549 cells

**Figure 3:**
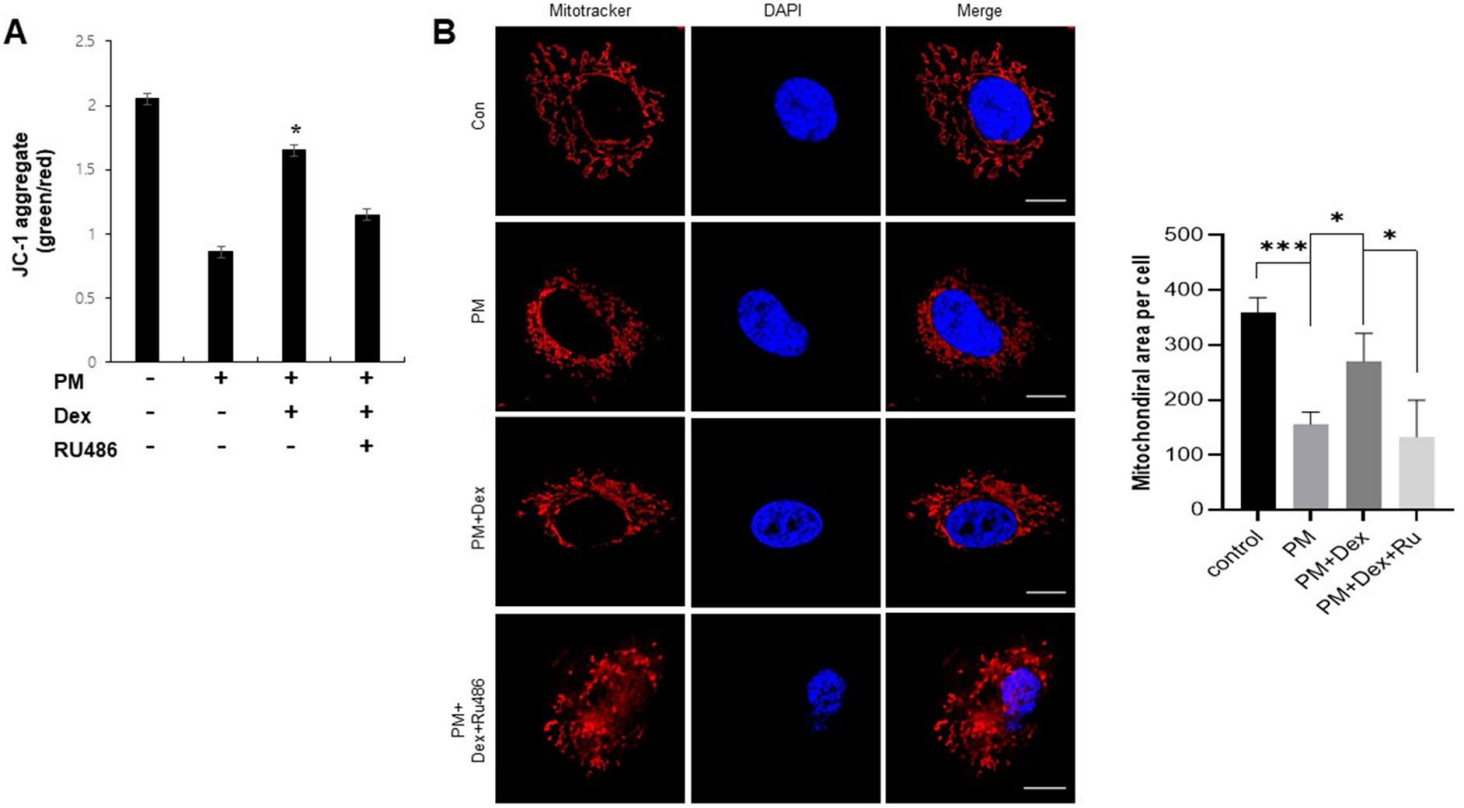
Glucocorticoids alleviate PM-induced mitochondrial dysfunction in A549 cells. (**A**) A549 cells were treated with PM (300 µg/ml) as indicated for 24 h and stained with JC-1. (**b**) A549 cells were treated with PM (300 µg/ml) as indicated for 24 h and stained with MitoTracker. Values represent the mean ± SEM (N = 3). *P < 0.05, ***P < 0.005. All experiments were repeated three times.

PM impairs mitochondrial function^33,34^. Glucocorticoids regulate mitochondrial function in a GR-dependent manner^24^. To evaluate the biological relevance of GR-mediated COX-2 regulation in PM-treated A549 cells, we measured the MMP using JC-1 staining and examined mitochondrial morphology using mitochondrial staining. We found that MMP dysfunction and mitochondrial fragmentation were induced during PM exposure compared to those during normal conditions, and dexamethasone significantly restored MMP dysfunction and mitochondrial fragmentation (Fig. 3A,B). These results suggested that glucocorticoids attenuate mitochondrial dysfunction in A549 cells exposed to PM.

### Glucocorticoid increases Bcl-2/GR complex formation in PM-treated A549 cells

**Figure 4:**
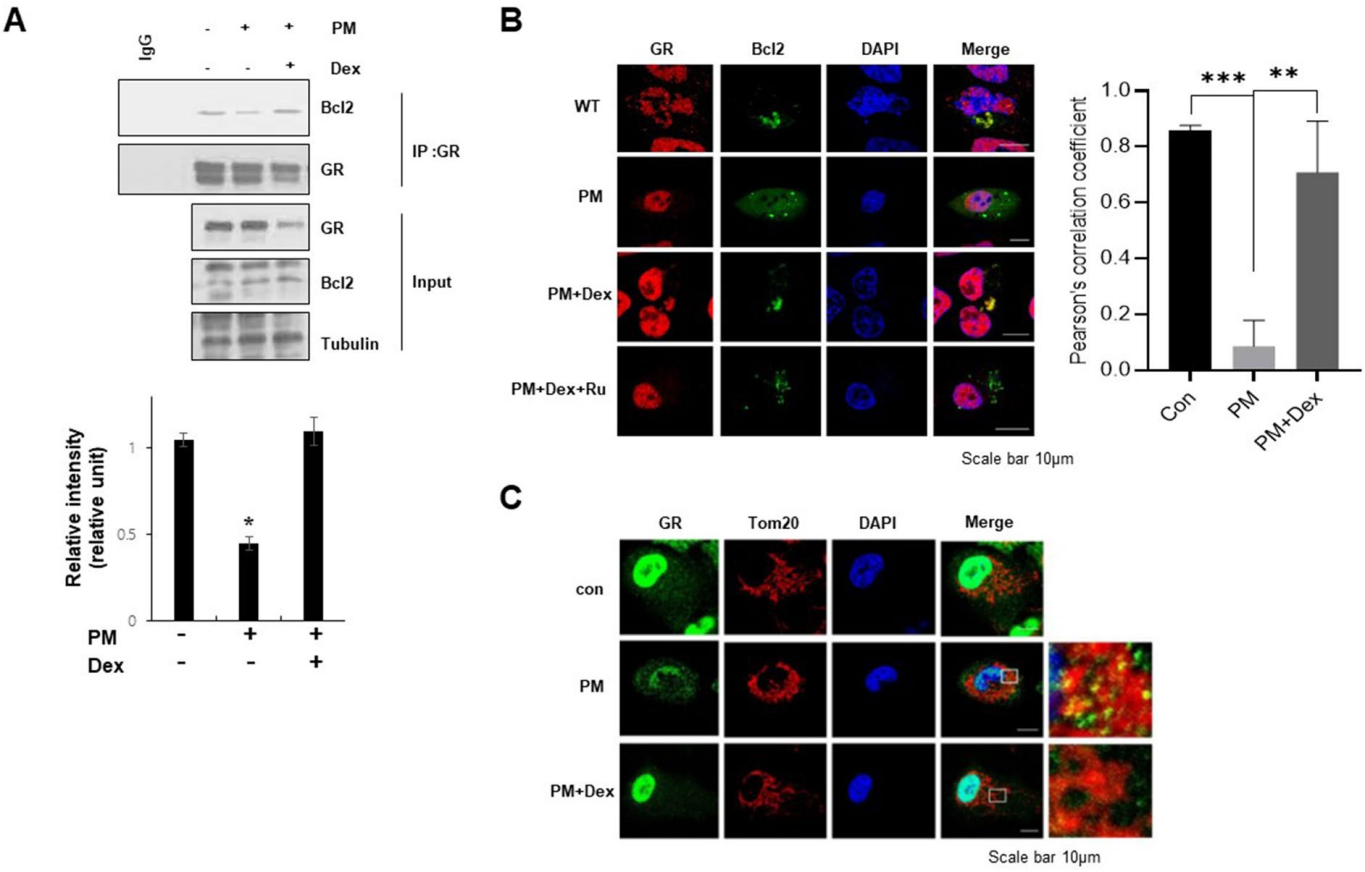
Glucocorticoid increases Bcl-2/GR complex formation in PM-treated A549 cells. (**a**) The whole cell lysates were immunoprecipitated with GR antibody, and western blot was performed with Bcl2 or GR antibody after immunoprecipitation. (**b**) A549 cells were treated with PM (300 µg/ml) as indicated for 24 h and immunostaining was followed using anti-GR or Bcl2 antibody. (**c**) Cells were stained with Tom20, and immunostaining was followed using anti-GR antibody. The inset shows GR on the mitochondria. Values represent the mean ± SEM (N = 3). *P < 0.05, **P < 0.005, ***P < 0.001. All experiments were repeated three times. The images of the original blots are available in Supplementary Fig. 2.

GR-mediated regulation of PM-induced mitochondrial dysfunction prompted us to investigate whether dexamethasone regulates Bcl-2/GR complex formation when exposed to PM. Co-IP assays were performed on PM-treated A549 cells. PM treatment significantly decreased the interaction between GR and Bcl-2, and glucocorticoids significantly restored Bcl-2/GR complex formation and colocalization, particularly in the mitochondria of PM-treated A549 cells (Fig. 4A,B). The localization of GR within PM-treated mitochondria was confirmed via immunocytochemical microscopy (Fig. 4C and Supplementary Fig. 6). This indicates that glucocorticoids regulate the physical interaction between GR and Bcl-2 during PM exposure.

## Discussion

In this study, there were two key findings: (1) GR suppressed PM-mediated lung inflammation in pulmonary epithelial cells via a mitochondrial ROS-dependent pathway, and (2) the protective role of GR in PM-induced lung inflammation was associated with the regulation of mitochondrial dysfunction and Bcl-2/GR complex formation. Impaired GR dimerization aggravates pulmonary dysfunction^35^, and GR-mediated apoptosis in SCLC requires Bcl-2/GR complex formation^22^. Proapoptotic Bcl-2 proteins are critical for PM-induced cell death and lung inflammation^36^. The Bcl-2 inhibitor reduced PM-induced lung inflammation by inducing neutrophil apoptosis^37^. However, the interaction between PM and GR-mediated anti-inflammatory processes has not been elucidated. Our data suggested that Bcl-2/GR complex formation is a key event in mediating the suppressive effect of glucocorticoids on PM-induced COX-2 expression.

A novel finding in this study was the identification of the role of GR/mitochondria/COX-2 pathway in PM-induced lung inflammation. This study further demonstrated the importance of GR and mitochondria in PM-treated SCLC as suppressive regulators of ROS and inflammatory responses. It seems that mitochondrial targeting of GR triggers at least two critical signals, ROS and Bcl-2, when exposed to PM. A previous study showed that the regulation of mitochondrial ROS by melatonin is mediated by GR^38^, which forms a complex with Bcl-2^24^. Consistent to this, our results revealed that elevated mitochondrial ROS and PM-induced decrease in Bcl-2/GR complex formation may serve as important mitochondrial signals for PM-induced COX-2 expression. Additional studies are required to identify the exact mechanisms underlying PM-induced ROS production in GR/Bcl-2 interactions at multiple levels.

PM exposure can increase lung inflammation and cancer^39,40^. Because COX-2 has a link between inflammation and cancer, COX-2 is a prime target for cancer^41^. Most cancers exhibit inflammation, characterized by increased production of prostaglandins^42^. PM has been shown to increase COX-2 expression and prostaglandin production^43–45^. These observations indicate the significance of inhibiting COX-2 and/or prostaglandins to prevent PM-induced lung injury and inflammation. There was no direct observation of the relationship between GR, mitochondria, and COX-2/PGE2 pathway during PM exposure. Hence, this study is the first to demonstrate that GR inhibits the PM-induced COX-2/PGE2 pathway through mitochondrial ROS and that the Bcl-2/GR complex could be critical for inhibiting PM-mediated lung inflammation and cancer progression.

## Conclusions

In conclusion, our results demonstrated that glucocorticoids ameliorated PM-induced lung inflammation by scavenging ROS, decreasing mitochondrial dysfunction, and restoring Bcl-2/GR complex formation in a GR-dependent manner. GR may be a key therapeutic target for suppressing inflammation caused by airborne particulate matter.

### Supplementary Information

Supplementary Figures.

## Data Availability

The datasets. Used and/or analyzed during the current study are available from the corresponding author on reasonable request.
