# Supplementary material for: Glucocorticoids alleviate particulate matter-induced COX-2 expression and mitochondrial dysfunction through the Bcl-2/GR complex in A549 cells
Source: Sci Rep. 2023 Nov 2;13:18884. doi: 10.1038/s41598-023-46257-y (PMC10622527; doi:10.1038/s41598-023-46257-y)

**Glucocorticoids alleviate particulate matter-induced COX-2 expression and mitochondrial dysfunction through the Bcl-2/GR complex in A549 cells**

Yeon-Ji Park^1, †^, June Heo^1, †^, Yonghyeon Kim^1^, Hyeseong Cho^1^, Myeongkuk Shim^2^, Kyunghyun Im^2^ and Wonchung Lim^3, *^

^1^Department of Biochemistry and Molecular Biology, Ajou University School of Medicine, Suwon, Republic of Korea; Department of Biomedical Sciences, Graduate School of Ajou University, Suwon, Republic of Korea.

^2^BL Healthcare, Yongin-si, Gyeonggi-do, 16827, Korea, South Korea

^3^Department of Sports Medicine, College of Health Science, Cheongju University, Cheongju, 28503, South Korea

*Corresponding author

Wonchung Lim, Ph.D.

Department of Sports Medicine, College of Health Science, Cheongju University, Cheongju, 28503, South Korea E-mail: wonchlim@gmail.com; Telephone: 82-043-229-8629

Supplementary Figure 1. The representative immunoblots and their multiple exposure images for Fig. 1. Cells were pretreated with dexamethasone (0.1 µM) and/or RU486 (1 µM) for 1 h before treatment with PM for 24 h and analyzed by Western blot.

Supplementary Figure 2. The representative immunoblots and their multiple exposure images for Fig. 4. The whole cell lysates were immunoprecipitated with GR antibody, and western blot was performed with Bcl2 or GR antibody after immunoprecipitation.

Supplementary Figure 3. GR knockdown reduces glucocorticoid inhibition of PM-induced COX-2 expression. A549 cells were transfected with siRNA targeting GR and pretreated with dexamethasone (0.1 μM) for 1 h before treatment with PM for 24 h. Immunoblots were probed with a COX-2 antibody or GR antibody

Supplementary Figure 4. (A549, GRE dependent gene transcription assay) A549 cells were transfected with GRE-Luc and treated as indicated. After treatment, luciferase expression was determined.

Supplementary Figure 5. (A549, positive control for COX-2 protein band) Cells were pretreated with dexamethasone (0.1 µM) and/or RU486 (1 µM) for 1 h before treatment with PM for 24 h and analyzed by Western blot. NaCl treatment is used for positive control for COX-2 induction.


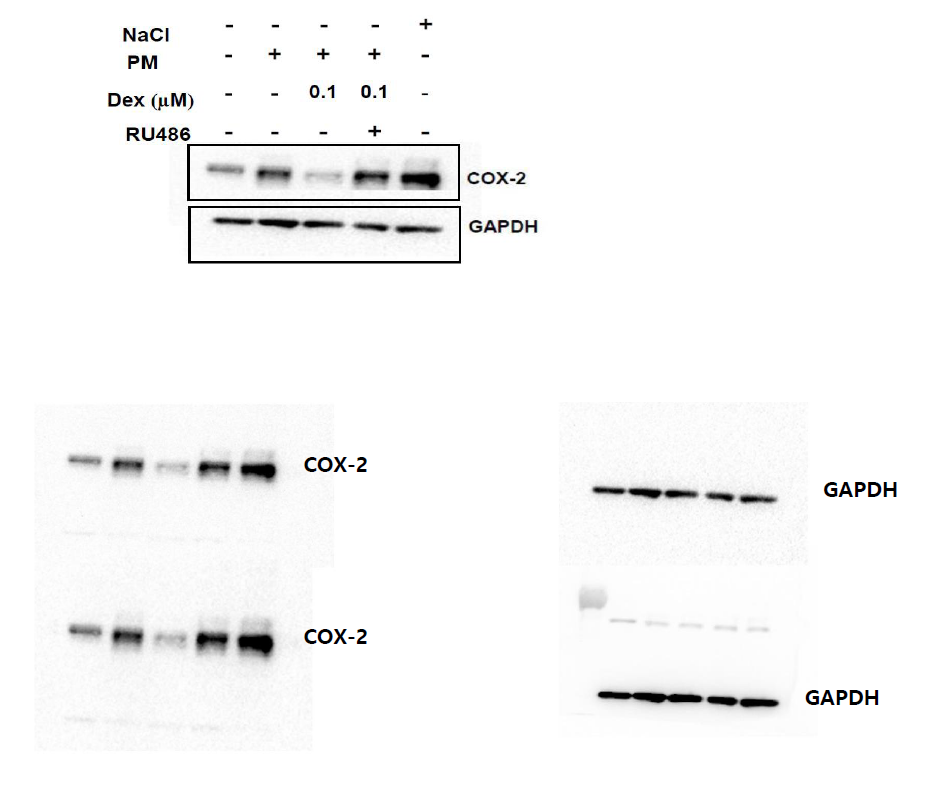


Supplementary Figure 6. (A549, cytosol fraction data for GR) A549 cells were treated as indicated, and cytosol and nuclear fractions were isolated. Identification of GR localization was measured by immunoblotting.


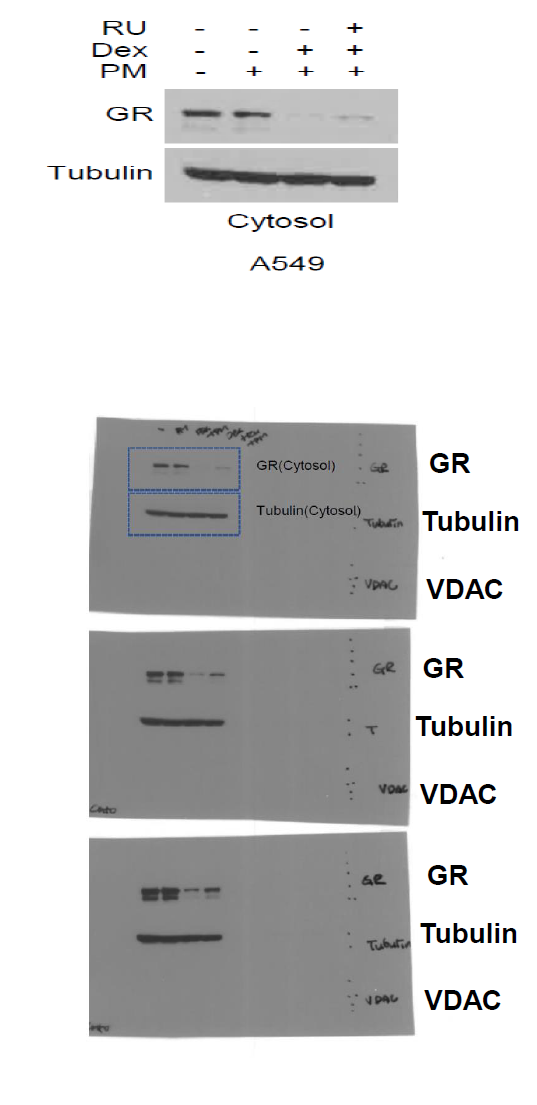

Supplement: Supplementary file 1 — Supplementary Figures. [file 41598_2023_46257_MOESM1_ESM.docx]
